# Improving stability of frying oils and food quality with addition of dried olive mill wastewater

**DOI:** 10.1038/s41538-025-00430-x

**Published:** 2025-05-16

**Authors:** Rokaya H. Jeba, Hanaa M. Hemada, Abdel Aziz Nadir, Mohamed E. Mansour

**Affiliations:** 1 Nutrition and Food Science Department, Faculty of Home Economics, Helwan University, Helwan, Egypt; 2 Food Technology Department, National Research Centre, Giza, Egypt

**Keywords:** Plant sciences, Health care, Agriculture

## Abstract

The olive oil industry in Mediterranean countries generates large quantities of waste, recognized as a cheap source of valuable compounds. This study evaluated the antioxidant properties, nutritional value and antimicrobial activity of dried olive mill wastewater (OMWW) and its effect on vegetable oils (corn and soybean) during frying and selected food products. OMWW was found to contain 5.75 g GAE/L of free phenols, with oleuropein being the most bioactive compounds. Refined vegetable oils enriched with OMWW (600 mg/L) showed an increase in induction times, indicating higher oxidative stability compared to oils with BHT. Sensory evaluation revealed no significant differences (*p* < 0.05) in characteristics of French fries which fried in enriched refined oils versus controls. OMWW polyphenols effectively retarded lipid oxidation and improved oxidative stability of oils, nutritional value of French fries, and sensory attribute of mayonnaise. Beneficial use of OMWW as a natural antioxidant was recommended by the present study.

## Introduction

The oxidation of vegetable oils poses a significant challenge by diminishing their sensory, nutritional and commercial quality. The resistance of oils to oxidation is influenced by both intrinsic and extrinsic factors. Key intrinsic factors include the composition of fatty acids and the presence of antioxidants, while major extrinsic factors encompass exposure to light, heat and air. The levels and effectiveness of natural antioxidants in vegetable oils vary greatly, influenced by the genotype of the source material, extraction techniques and refining processes. Often, natural antioxidants alone are insufficient to prevent oxidation, necessitating the addition of external antioxidants to enhance oil stability^1^.

Frying is a widely utilized method for food preparation prized for its speed, convenience and ability to produce flavorful and appealing foods. Frying uses oil as an efficient heat transfer medium. The chemical reactions during frying occurring at a faster rate, result in the rapid decomposition of unstable hydroperoxides into secondary oxidation products. Therefore, antioxidant is considered to be one of the most common ways to limit the oxidation process^2^. The interest of researchers^3^ and food manufacturers in polyphenols has surged, primarily due to it is significant antioxidant properties that provide a delay in the oxidative process. Therefore, it should be abundance in food, and their potential role in preventing diseases linked to oxidative stress and inflammation, including cancer, diabetes, cardiovascular and neurodegenerative diseases.

Vegetable oils such as sunflower, corn and soybean oils often include the synthetic antioxidant beta hydroxy acids (BHA) to extend their oxidative stability due to their unsaturated fatty acids and low antioxidant content, making them prone to oxidation and off-flavors. Replacing synthetic antioxidants with natural alternatives is a major focus of numerous studies in this field^4^. Chiou et al. enhanced oxidative stability of commercially available sunflower, olive and refined palm oils by adding olive leaf extract (OLE) and observed their performance during pan-frying. This addition increased antioxidant levels by 1.5–6.9 times in sunflower oil, 1.1–2.5 times in olive oil and 1.9–3.8 times in palm oil. Despite loss of phenolic compounds during frying, the polyphenolic content in French fries corresponded to the level of oil supplementation^5^.

The olive tree (*Olea europaea L*.) is extensively cultivated in the Mediterranean region, thriving in its mild climate^6^. Olive oil extraction produces olive oil (20%), semi-solid waste (30%) and olive mill wastewater (OMWW) (50%), which contains mainly water as by a product of the olive processing. Most olive phenols prefer the water phase, with partition coefficients ranging from 6 × 10^−4^ to 1.5. According to Gueboudji et al. ^7^, while olive fruit is rich in phenolic compounds, only 2% of these pass into the oil phase, with about 53% lost in OMWW and remains in the pomace. Both by-products, particularly OMWW, are environmentally harmful, affecting soil microbial populations. Annual global production of OMWW is estimated between 7 and over 30 million m^3^, with total phenolic compounds up to 22.97 g/100 g and over 50 phenolic molecules, depending on the type of olive mill and equipment used. The OMWW could serve as an economical and natural antioxidant source due to its “high phenolic content in their preferred aqueous environment”. Ribeiro et al. ^8^ assessed the safety of olive pomace powder (OP) at concentrations ranging from 10 to 1000 μg/plate, finding no cytotoxicity or mutagenicity, suggesting potential security at these levels.

In this context, the present study aimed to identify the main bioactive compounds in OMWW and evaluate the potential of dried OMWW as a natural antioxidant in vegetable oils. The investigation sought to enhance the oxidative stability and shelf life of refined vegetable oils, as well as improve the sensory properties of foods high in vegetable oils, such as French fries and mayonnaise. Additionally, the study assessed the nutritional value of the enriched mayonnaise.

## Results and discussion

### Physiochemical characteristics of OMWW

The polyphenolic composition of dried olive mill wastewater was identified through HPLC analysis in the present study, as shown in (Table 1). The results indicated that concentrations of bioactive compounds, comprised: oleuropein, protocatechuic acid, *p*-hydroxybenzoic acid, chlorogenic acid, syringic acid, caffeic acid, p-coumaric acid and vanillic acid, were as the follows: 2.35, 0.66, 0.10, 0.08, 0.05, 0.04, 0.02, and 0.01 mg/g, respectively. As shown in (Table 1) among the entire polyphenol compound presented in dried olive mill waste water, oleuropein concentration was the highest. As stated by Karković Marković et al. ^9^, all these phenolic compounds are considered bioactive and possess various beneficial properties, including antioxidant, antitumor, antimicrobial, antiviral and anti-inflammatory effects.

**Table 1:** Polyphenolic composition, physiochemical determinations, antioxidant activity DPPH and antimicrobial activity, of dried olive mill waste water

Parameter	OMWW	Antioxidant activity DPPH		Antimicrobial activity of OMWW		
1-Polyphenolic composition: Oleuropein (mg/g)	2.35	Sample	Inhibation %	Antimicrobial activity of OMWW	Inhibition zone diameter (mm/Sample)	Standard antibiotic
Protocatechuicacid (mg/g)	0.66	50 mg/kg	23.15 ± 0.08	**Gram negative bacteria**		Gentamicin
*p*-hydroxybenzoic acid (mg/g)	0.10	100 mg/kg	30.26 ± 0.10	*Escherichia coli*	13.3 ± 0.5	27 ± 0.5
Chlorogenic acid (mg/g)	0.08	150 mg/kg	40.14 ± 0.11	*Klebsiella pneumonia*	12.0 ± 1.0	25 ± 0.5
Syringic acid (mg/g)	0.05	200 mg/kg	59.14 ± 0.22	*Pseudomonas aeruginosa*	NA	28 ± 1.0
Caffeic acid (mg/g)	0.04	400 mg/kg	70.12 ± 0.25	**Gram positive bacteria**		Ampicillin
*p*-coumaric acid (mg/g)	0.02			*Staphylococcus aureus*	NA	22 ± 0.1
Vanillic acid (mg/g)	0.01			*Streptococcus mutans*	NA	30 ± 0.5
**2- pH of OMWW** pH	5.25 ± 0.59			**Fungi**		Nystatin
**3- Total soluble solids** TSS g/L	10.7 ± 0.36			*Candida albicans*	10.0 ± 1.0	21.0 ± 1.0
FP g GAE/L	5.75 ± 0.31			*Asperagillus niger*	NA	19.0 ± 0.7
TF g CAE/L	2.42 ± 0.25			*Asperagillus Ochraceous*	NA	15 ± 1.0

Data were presented as (mean ± SD) except data of antimicrobial activity were presented as (mean ± SE).

*OMWW* olive mill wastewater. *NA* No activity.

These findings align with the results of study carried out by Benincasa et al. ^10^, who reported that phenolic compounds identified in dried olive mill wastewater included oleuropein, caffeic acid, p-coumaric acid and vanillic acid, with concentrations of 0.103, 0.003, 0.005 and 0.028 mg/g, respectively. However, the concentration of oleuropein of the present study 2.35 mg/g was found to be higher than the value 0.013 mg/g reported by Benincasa et al. ^10^. This variation could be due to differences in the variety of olive mills and storage conditions before drying. Also, the drying temperature in the present study was 40 °C, whereas the previous study was 165 °C. The high temperature might have contributed to the loss of phenolic compounds.

Physicochemical parameters of dried OMWW are presented in Table 1. OMWW in the present study exhibited an aroma reminiscent of olive fruit, with a slightly acidic pH of 5.25 which found to be a little higher than the value reported pH of 4.7 by ref. ^11^ and close to the value 5.05 reported by Gueboudji et al. ^12^.

The present study showed that total soluble solids (TSS) content was measured at 10.7 g/L, was higher than the range of 2.1–4.5 g/L reported by ref. ^13^. Free phenol content (FP) and total flavonoid content (TF) reported in the present study were 5.75 g GAE/L and 2.42 g CAE/L, respectively. The value of FP obtained in the present study was lower than value FP: 7.9 g GAE/L reported by Romeo et al. ^14^. However, FP result was higher than that values 1.34 g GAE/L reported by ref. ^15^ and lower than the reported values 6.02 g GAE/L by De Bruno et al. ^16^. The value of TF obtained in the present study was lower than value 9.83 g CAE/L reported by Alrowais et al. ^17^.

The results of Hunter colorimeter analysis of dried OMWW (Table 2) revealed a lightness (*L*) value of 92.12, redness (*a*) value of 0.02 and a yellowness (*b*) value of 2.32. These results agreed with those values *L* = 33.82, *a* = 0.45 and *b* = 3.24 reported by ref. ^18^. Mineral and vitamin content of OMWW (Table 2), indicated that the values for calcium, phosphorus, potassium and iron were found to be 215.0, 280.0, 1607.5 and 31.25 mg/100 g, respectively. Different values of minerals were reported 142.1, 203.4 and 4400 mg/100 g respectively by ref. ^19^. The OMWW content of vitamins E and A in the present study were 1 mg/100 g and 1.12 IU/g, respectively. While, the study done by Aggoun et al. ^20^, tocopherol and vitamin A content of OMWW (from different species and extraction systems), reported to be 1.85–4.47 mg/100 g and 0.23–0.96 mg/100 g, respectively. Tocopherols play a crucial role in protecting against the oxidation of polyunsaturated fatty acids and the free radical oxidation of biological materials and lipoproteins, serving as important lipid antioxidants as stated by ref. ^21^

**Table 2:** Hunter colorimeter, minerals and vitamins content of dried olive mill waste water

Parameter	Unit	OMWW
**Hunter colorimeter**		
*L*	-	92.12 ± 0.33
*a*	-	0.02 ± 0.01
*b*	-	2.32 ± 0.21
**Minerals and vitamins content**		
Calcium	mg/100 g	215.0 ± 3.60
Phosphorus	mg/100 g	280.0 ± 2.65
Potassium	mg/100 g	1607.5 ± 1.41
Iron	mg/100 g	31.25 ± 1.00
Tocopherol vitamin	mg/100 g	1 ± 0.30
A vitamin	IU/g	1.12 ± 0.28

Data were presented as (mean ± SD).

*OMWW* olive mill wastewater.

It was observed that dried (OMWW) exhibits hygroscopic properties. This characteristic may be attributed to the presence of various minerals, particularly those with specific chemical compositions or crystal structures that possess hygroscopic qualities^22^. The variations in proximate composition of OMWW across different studies can be attributed to several factors, including cultivar differences, climatic conditions, soil type, fertilizers used, irrigation methods, olive varieties, fruit ripeness, harvest time and the extraction processing system^23^.

### Antioxidant activity of OMWW

The antioxidant activity of dried OMWW at different concentrations is summarized in (Table 1). Results demonstrated an increase in antioxidant activity with higher concentrations of OMWW 50 mg/kg, 100 mg/kg, 150 mg/kg, 200 mg/kg, and 400 mg/kg, yielding values of 23.15, 30.26, 40.14, 59.14, and 70.12 μg/mL, respectively. El Moudden et al. investigated the property of OMWW from various sources for their antioxidant effect and reported values ranging from 0.32 to 1.93 μg /mL^24^. Similarly, ref. ^11^ studied different concentrations of OMWW extract up to 1600 mg/kg, with IC_50_ values reaching 118 μg /mL, attributing this activity to phenolic compounds, flavonoids and vitamin E present in OMWW.

The observed increase in antioxidant activity with concentration supports the concept that OMWW includes powerful antioxidants capable of reducing oxidative stress, which is essential in biological systems to fight the damaging effects of free radicals. OMWW contains a high concentration of phenolic chemicals, especially oleuropein, which is known for its antioxidant properties.

According to study, these chemicals can effectively neutralize free radicals and minimize oxidative damage, resulting in the observed dose-dependent increase in antioxidant activity. These data indicate that, when administered at adequate quantities, OMWW can be an important source of antioxidants. This proposal supports continuing research on using olive mill waste by-products as natural antioxidants in industries such as food, cosmetics and medicines^25^.

### Antimicrobial activity

The present study measured the diameters (mm) of the inhibition zones (size of the halo formed) and the results presented in (Table 1). The findings indicated that OMWW exhibited an inhibitory effect against the gram-negative bacteria *Escherichia coli* and *Klebsiella pneumoniae*, with inhibition zones measuring 13.3 mm and 12 mm, respectively. These results were consistent with those reported by Afonso et al.^26^. In contrast, OMWW showed no inhibitory effect against the gram-positive bacteria *Staphylococcus aureus* and *Streptococcus mutans*. Additionally, the inhibitory effect of OMWW against fungi was 10.0 mm for *Candida albicans*.

### Effect of dried OMWW on oxidative stability of vegetable oils using the Rancimat method

The Rancimat method accelerates the oxidation process by maintaining a high temperature of 100 °C and a continuous stream of air applied to the oil samples^27^. Induction time refers to the duration required to reach an endpoint of oxidation, which corresponds to a rapid change in the oxidation rate. In the present study, the stability of enriched corn oil at concentrations of 200, 400, and 600 mg/L was evaluated against the control sample (without additives) and sample with butylated hydroxyl toluene (BHT) (200 mg/L). The results of the present study showed that the induction time for the control sample was (12.30 h), while the induction time increased to 13.53 h for sample with BHT (200 mg/L) sample. The enriched corn oil samples with OMWW demonstrated an increase in induction period with rising OMWW concentrations: 13.41 h for 200 mg/L, 14.19 h for 400 mg/L and 15.10 h for 600 mg/L, respectively. These findings indicate the antioxidant property of dried OMWW, which extended the shelf life of enriched corn oil compared to the other samples.

The results of the present study were consistent with those of ref. ^28^, who examined the effect of OMWW extract at 100 mg/L on various vegetable oils. The investigators stated that OMWW extract retarded the oxidation process of sunflower and rapeseed oils, thereby extending their shelf life. Additionally, the findings align with those of ref. ^14^, who studied the impact of phenolic extracts at 50 mg/L of oil from OMWW on the oxidation stability of sunflower oil. In contrast, M’rabet et al. found that peroxide value (PV) of sunflower oil supplemented with 0.25 and 0.5% with oleuropein-rich olive leaf (OLE) revealed decrease in PV compared to control sunflower oil without OLE^29^.

The data in (Table 3) shows the oxidative stability results of soybean oil, the induction times for oil samples enriched with different concentrations of dried OMWW 200, 400 and 600 mg/L increased to 12.21, 12.93 and 13.63 h, respectively, compared to the control sample 11.61 h. The induction time of enriched sample with high concentration 600 mg/L was greater than that of BHT 200 mg/L sample, which had induction time of 13.50 h. These findings indicated that dried OMWW contained bioactive compounds that act as antioxidants, including polyphenols especially in the present of high concentration of oleuropein, flavonoids, carotenoids, and tocopherols. The synergistic effect of antioxidant components presented in Tables 1, 2 the dried OMWW led to oxidative stability and extent the shelf life of enriched corn oil and soybean oil compared to synthetics antioxidant BHT and control (without antioxidant).

**Table 3:** Oxidative stability of corn oil and soy bean oil as affected by different concentrations of dried OMWW using Rancimat method

Sample	Induction time (h)	Antioxidant activity	Increasing index (%)
	**Corn Oil**		
Control	12.30 ± 0.40	-	-
BHT (200 mg/L)	13.53 ± 0.20	1.10	10.0
OMWW (100 mg/L)	12.40 ± 0.20	1.01	0.08
OMWW (200 mg/L)	13.41 ± 0.15	1.09	9.02
OMWW (400 mg/L)	14.19 ± 0.09	1.15	15.37
OMWW (600 mg/L)	15.10 ± 0.30	1.23	22.76
	**Soybean Oil**		
Control	11.61 ± 0.15	-	-
BHT (200 mg/L)	13.50 ± 0.14	1.16	16.28
OMWW (200 mg/L)	12.21 ± 0.08	1.05	5.168
OMWW (400 mg/L)	12.93 ± 0.18	1.11	11.37
OMWW (600 mg/L)	13.63 ± 0.19	1.17	17.40

Data were presented as (mean ± SD).

*OMWW* olive mill wastewater.

### Sensory evaluation results of French fries and mayonnaise

The sensory evaluation results (scores) for French fries prepared in enriched oils with dried OMWW are shown in (Table 4). The results indicated no significant differences existed (*p* < 0.05) in sensory scores between potato fried in enriched oil samples and the control samples across all sensory characteristics. These findings were consistent with what reported by Chiou et al. ^5^, who indicated that panelists found acceptable sensory attributes, including taste, color and crispiness. This could likely be attributed to the low concentration of dried OMWW used 600 mg/L. The findings of the present study may encourage food industries to use dried OMWW in suitable concentration as alternative to the synthetic antioxidants.

**Table 4:** Mean values of sensory characteristics of French fries and mayonnaise prepared with enriched dried olive mill wastewater, as well as their free phenol content

^A^Sensory characteristics of French fries						
Characteristics	Appearance	Odor	Taste	Color	Tenderness	General acceptability
**Control** ^**(−)**^	4.8 ± 0.42^a^	4.70 ± 0.67^a^	4.70 ± 0.64^a^	4.80 ± 0.42^a^	4.50 ± 0.48^a^	4.70 ± 0.42^a^
**Control** ^**(+)**^	4.8 ± 0.41^a^	4.75 ± 0.52^a^	4.80 ± 0.42^a^	4.80 ± 0.42^a^	4.60 ± 0.55^a^	4.75 ± 0.44^a^
**600** **mg/L OMWW**	4.55 ±0.64^a^	4.60 ± 0.42^a^	4.60 ± 0.39^a^	4.55 ± 0.50^a^	4.65 ± 0.67^a^	4.80 ± 0.42^a^
^A^**Sensory characteristics of mayonnaise**						
**Characteristics**	**Odor**	**Taste**	**Flavor**	**Color**	**Degree of cohesion**	**Degree of softness**
**Control** ^**(−)**^	4.35±0.47^a^	4.45 ± 0.50^a^	4.50 ± 0.47^a^	4.60 ± 0.46^a^	4.80 ± 0.35^a^	4.85 ± 0.33^a^
**Control** ^**(+)**^	4.65 ± 0.47^a^	4.55 ± 0.48^a^	4.50 ± 0.53^a^	4.40 ± 0.51^a^	4.75 ± 0.42^a^	4.65 ± 0.47^a,b^
**1% OMWW**	3.90 ± 0.21^b^	3.80 ± 0.26^b^	3.90^b^	4.00 ± 0.11^b^	4.40 ± 0.46^a^	4.45 ± 0.44^a,b^
**3% OMWW**	3.30 ± 0.35^c^	3.40 ± 0.41^c^	3.35 ± 0.54^c^	4.30 ± 0.48^c^	4.35 ± 0.58^a^	4.35 ± 0.51^b^
^B^**Free phenol content (mg/100** **g GAE) of French fries and mayonnaise prepared with enriched dried olive mill wastewater**						
**Products**	**Control** ^**(−)**^			**600** **mg/L OMWW**		
**French fries (mg/100** **g GAE)**	52.17 ± 0.24^b^			54.61 ± 0.25^a^		
**Mayonnaise (mg/100** **mL GAE)**	1.99 ± 0.12^b^			11.33 ± 0.07^a^		

^A^Different lowercase letters in the same column indicate that there are statistically significant differences between the means, with a significance level of *P* < 0.05. Data were presented as (mean ± SD)

*OMWW* olive mill wastewater, Control (–) is referring to French fries sample with no added antioxidants, Control (+) is referring to French fries sample with TBHQ (200 mg/L). (*p* < 0.05).

^B^Different lowercase letters in the same row indicate that there are statistically significant differences between the means, with a significance level of *P* < 0.05. Data were presented as (mean ± SD)

*OMWW* olive mill wastewater. Control (–) is referring to French fries sample with no added antioxidants, The level of significant is (*p* < 0.05).

^a,b,c^indicate statistical differences between values.

Results in Table 4 showed that odor, taste, flavor, color and general acceptability showed significant differences at (*p* < 0.05) between the enriched sample and the control samples. There was a decrease in sensory scores as the levels of dried OMWW increased. While there were no differences significant difference was found at (*p* < 0.05) between the enriched sample and control samples in a degree of cohesion characteristics. On the other hand, the score for softness decreased in enriched sample compared to control ^(-)^. The results of the present study accordance with ref. ^30^, who used phenol extract from OMWW in mayonnaise preparation and found an increase in bitterness in PE samples compared to control. Also, color values of phenol extract mayonnaise decreased compared to control. De Leonardis et al. ^31^ illustrated that effect of olive leave in mayonnaise on textural of mayonnaise samples were homogenized. Although color of olive leave in mayonnaise was light brown and taste had a bitterness aftertaste duo to the present of oleuropein in olive leave in mayonnaise.

### Free phenol content of French fries and mayonnaise

Results of the free phenol content of French fries prepared with enriched dried OMWW are presented in Table 4. Results showed dried OMWW at 600 mg/L have a significant content (*p* < 0.05) higher in free phenol content than compared to the control. The results agreed with the study done by Chiou et al. ^32^, who found that the free phenol content of French fries with olive leaf extract higher than that of French fries treated without olive leaf extract after frying. Also, the present study showed that the enriched sample of mayonnaise with 3% dried OMWW has the highest significant content (*p* < 0.05) of free phenol compared to the mayonnaise sample without OMWW, which agreed with the results of other investigators^30^.

### Thiobarbituric acid (TBA) value of French fries and mayonnaise

**Fig. 1:**
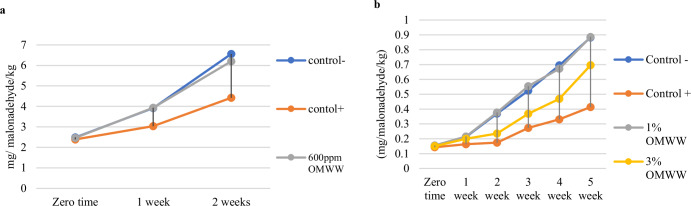
Thiobarbituric acid values. **a** TBA value of French fries. **b** TBA value of mayonnaise. The results are under storage conditions. OMWW: olive mill wastewater, Control (–) is referring to mayonnaise sample with no added antioxidants, Control (+) is referring to mayonnaise sample with TBHQ (200 mg/L). The level of significant is (*p* < 0.05).

Thiobarbituric acid (TBA) values for French fries prepared with and without dried OMWW during storage (at 25 ± 1 °C for 2 weeks) are presented in (Fig. 1). The results indicated no significant differences (*p* < 0.05) in TBA values between the enriched and control samples at zero time. However, after 2 weeks, the TBA value of the enriched sample with 600 mg/L OMWW showed significant differences (*p* < 0.05) and was reduced compared to the control sample without antioxidants treatment due to an increased in free phenol content of French fries treated. A previous study done by ref. ^33^ noted that using PE from OMWW in frying potatoes in olive oil helped protect the fried food and oil from oxidative degradation compared to the control without added PE.

Results of thiobarbituric acid for enriched mayonnaise samples with different levels of dried OMWW during storage at 25 ± 1°C for 5 weeks are presented in (Table 5). Results indicated that TBA value of the enriched sample with 3% OMWW had significant differences at (*p* < 0.05) with the control ^(−)^ sample at zero time and during a storage period for 5 weeks at room temperature. The antioxidant activity of dried OMWW retards the lipid peroxidation in the mayonnaise sample 3% OMWW. Results of the present study agree with the findings of De Leonardis et al. ^31^ who reported that the effect of adding 8% of olive leaf vinegar in mayonnaise had oxidative stability during storage period compared to control but after storage period for 12 months became low in peroxide value at olive leaf vinegar- mayonnaise. Also, De Bruno et al. ^30^ found that resistance to rancidity in enriched mayonnaise phenolic extracts was higher than that of control at the end of storage period.

### Total bacterial counts (TBC) of mayonnaise

Total bacterial count log cfu/g of enriched mayonnaise with different levels of dried olive mill wastewater during storage at 25 ± 1 °C for 5 weeks is presented in (Table 5). Results indicate that control mayonnaise and enriched samples with dried OMWW at zero time were contained low total bacterial counts (TBC) with value range of 2.28 to 2.30 log cfu/g. Results showed that total count bacterial values for enriched samples and control sample increased periodically through storage period. There was significant differences (*p* < 0.05) between samples enriched with 3% OMWW 4.68 cfu/g which found to be lower than control ^(−)^ 5.64 cfu/g in total bacterial counts after 5 weeks. While control ^(+)^ with TPHQ sample had the lowest 4.50 cfu/g bacterial counts compared with other investigated samples, but the result was close to 3% OMWW sample, it is possible that greater OMWW concentrations have some antibacterial activity, although it is still less potent than the synthetic antioxidant.

**Table 5:** Thiobarbituric acid, Total bacterial count, and Hunter color values of mayonnaise prepared supplemented with dried olive mill wastewater

Mayonnaise samples	Thiobarbituric acid (TBA) (mg/kg)			Total bacterial counts (TBC) log cfu/g			Hunter color values				
	Zero time	3week	5week	Zero time	3week	5week	L*	a*	b*	Saturation index	Hue angle
**Control** ^**(−)**^	0.15 ± 0.00^a^	0.53 ± 0.01^a^	0.88±0.02^a^	2.28 ± 0.04^a^	4.36 ± 0.09^a^	5.64 ± 0.11^a^	87.53 ± 0.36	0.71 ± 0.04	11.56 ± 0.32	11.58	86.46
**Control** ^**(+)**^	0.14 ± 0.00^b^	0.27 ± 0.01^c^	0.41±0.01^c^	2.30 ± 0.06^a^	3.20 ± 0.08^b^	4.50 ± 0.11^b^	87.88 ± 0.28	0.11 ± 0.03	10.85 ± 0.40	10.85	89.42
**1% OMWW**	0.16 ± 0.00^a^	0.56±0.01^a^	0.89±0.02^a^	2.30±0.06^a^	4.36 ± 0.11^a^	5.68 ± 0.14^a^	85.21 ± 0.34	1.07 ± 0.03	12.05 ± 0.19	12.10	84.93
**3% OMWW**	0.15 ± 0.00^a,b^	0.37±0.01^b^	0.70±0.02^b^	2.30 ± 0.06^a^	4.38 ± 0.11^a^	4.68 ± 0.14^a,b^	84.33 ± 0.13	1.12 ± 0.03	13.25 ± 0.25	13.30	85.17

Different lowercase letters in the same column indicate that there are statistically significant differences between the means, with a significance level of *p* < 0.05. *OMWW* olive mill wastewater, Control (–) is referring to mayonnaise sample with no added antioxidants, Control (+) is referring to mayonnaise sample with TBHQ (200 mg/L). Data were presented as (mean ± SD). L* Lightness. a* Redness. b* Yellowness.

Ref. ^34^ studied the effect of phenol extract at 400 and 600 mg/kg, from OMWW on the survival of *Salmonella Enteritidis* inoculated on mayonnaise. The investigators found that the OMWW phenolic extract exhibited an antibacterial impact, decreasing the growth rate of *Salmonella* by 9.5% at 22 °C and 5.8% at 4 °C per h within just 1 h of inoculation. It is worth noting that, ref. ^35^ suggest that use of phenol extract as a natural preservative agent is recommended to enhance the microbiological quality of mayonnaise. Collectively, these data show that integrating larger concentrations of OMWW in mayonnaise might not only aid in lowering bacterial counts but also serve as a viable alternative to synthetic preservatives, therefore boosting both safety and shelf life.

### Color evaluation of mayonnaise

Hunter colorimeter results of enriched mayonnaise with different levels of dried olive mill wastewater are presented in Table 5). It was found that lightness (L) decreased in enriched samples 1% OMWW and 3% OMWW compared to control ^(−)^ value 87.53. While redness (a) increased in enriched samples 1% OMWW and 3% OMWW with increased supplement proportion compared to control ^(−)^ value 0.71. Yellowness (b) of enriched samples increased compared to control ^(−)^. The results of the present study agreed with the finding reported by De Bruno et al. ^30^ who stated that the brown color increase with increased levels of OMWW. However, the present results indicated that saturation value for enriched mayonnaise with 1%OMWW and 3% OMWW decreased 12.10 and 13.30; respectively compared to control ^(−)^ value 11.58. Results indicated that saturation with brown color increases with increased concentration of OMWW.

### Nutritional value of Mayonnaise

The proximal composition of mayonnaise prepared with different levels of dried OMWW is presented in Table 6. It was found that no significant differences (*p* < 0.05) among the prepared samples in protein, fat and carbohydrate content. However, there were significant differences (*p* < 0.05) between all samples in ash content. Minerals composition of enriched mayonnaise with dried OMWW is presented in Table 6. It was found that calcium and potassium content in enriched sample with 3% OMWW were found to be higher 68 mg/100 g and 510 mg/100 g, respectively than that of control sample 44 mg/100 g and 103 mg/100 g, respectively. Results indicated that 3% OMWW enriched sample increased in its content of tocopherol 29.5 mg/100 g compared to control 23.5 mg/100 g. The increased content of calcium (Ca), phosphorus (P) and vitamin E in 3% OMWW mayonnaise sample can be primarily due to the mineral and vitamin content of olive mill wastewater (OMWW) as remembered in Table 2.

**Table 6:** Proximal composition % of mayonnaise prepared with different levels of dried OMWW

Samples	Protein	Fat	Carbohydrate	Ash	Ca (mg/100 g)	K (mg/100 g)	Tocopherol (mg/100 g)
**Control** ^(−)^	40.36 ± 1.83^a^	37.32 ± 1.69^a^	22.10 ± 5.92^a^	0.22 ± 0.01^a,b^	44 ± 2.65	103 ± 2.65	23.5 ± 1.73
**Control** ^**(+)**^	40.31 ± 1.76^a^	37.91 ± 1.72^a^	21.55 ± 0.96^a^	0.23 ± 0.01^a^	-	-	-
**1% OMWW**	39.08 ± 1.77^a^	37.08 ± 1.68^a^	23.62 ± 1.06^a^	0.22 ± 0.01^a,b^	-	-	-
**3% OMWW**	38.92 ± 1.76^a^	36.94 ± 1.67^a^	23.93 ± 1.07^a^	0.21 ± 0.01^b^	68 ± 2.65	510 ± 5.29	29.5 ± 3.28

Different lowercase letters in the same column indicate that there are statistically significant differences between the means, with a significance level of *p* < 0.05. OMWW: OMWW, Control (–) is referring to mayonnaise sample with no added antioxidants, Control (+) is referring to mayonnaise sample with TBHQ (200 mg/L). Data were presented as (mean ± SD).

The results of this study indicate that the addition of dried OMWW has a positive effect on the nutritional and physical properties of vegetable oils, improving their stability and reducing oxidation. The results also reported that the resistance to rancidity in mayonnaise enriched with dried OMWW was improved. The results also confirmed no significant differences in sensory characteristics between the samples with and without olive mill wastewater, suggesting its potential as a natural alternative in food processes. The conversion of this residual material into value-added products or applications plays a crucial role in enhancing the sustainability of the targeted resource. Further studies are recommended to explore the effects of higher concentrations of olive mill wastewater on the properties of other food products. Food industries should consider using olive mill wastewater as a natural additive to enhance the nutritional value and stability of oils in various cooking applications.

## Materials and methods

### Materials

Olive fruits (*Olea europaea* L.) from the Koroneiki cultivar were used in the study (the crop season of mid-November 2022 which was obtained from the Menoufia Governorate, Egypt). Refined corn oil (free fatty Acids 0.08%, density at 20 (0.920 g/ cm^3^)) and soybean oil (free fatty Acids 0.05%, density at 20 (0.922 g/ cm^3^)) (antioxidants free) were supplied from ARMA (10th of Ramadan city, Egypt). Ingredients for products preparation (french fries and mayonnaise), were purchased from the local market. All chemicals used in the study as well as BHT were purchased from Sky Chem Co., Cairo, Egypt. Gram (−) bacteria, gram (+) bacteria and fungi, were obtained from Faculty of Science, Cairo University, Egypt. El-Nasr Co., Cairo, Egypt, supplied the methanol, ethanol, dimethylsulfoxide (DMSO) and ampicillin.

### Methods

#### Preparation of dried OMWW

Olive oil and its by-products (olive mill wastewater and pomace) were produced using a machine that operates on a three-phase system. The method followed for OMWW preparation was according to Benincasa et al. ^10^. The OMWW obtained was dried and placed in an oven at 40 °C until dry, then packed in a plastic bottle to the full and sealed and stored at 4 °C until use.

### Physiochemical determination of dried OMWW

#### Polyphenol composition

The dried OMWW was subjected to HPLC analysis according to the method reported by ref. ^36^. A sample (1 g) was placed in a quick-fit conical flask and 20 mL of 2 M NaOH was added. The flasks were flushed with N_2_ and the stoppers were replaced. The samples were shaken for 4 h at room temperature. The pH was adjusted to 2 with 6 M HCl. The samples were centrifuged at 5000 rpm for 10 min and the supernatant was collected. To extract the phenolic compounds, the samples were subjected to two extractions using a mixture of 50 mL of ethyl ether and ethyl acetate in a 1:1 ratio. The organic phase underwent separation and evaporation at 45 °C, after which the resulting samples were dissolved again in 2 mL of methanol. Subsequently, HPLC analysis was conducted using an Agilent Technologies 1100 series liquid chromatograph featuring an auto-sampler and a diode-array detector (Agilent Technologies, California, USA). The analytical column was an Eclipse XDB-C18 (150 × 4.6 mm; 5 µm) with a C18 guard column (Phenomenex, Torrance, California, USA.). The mobile phase consisted of acetonitrile (solvent A) and 2% acetic acid in water (v/v) (solvent B). The flow rate was maintained at 0.8 mL/min for a total run time of 70 min and the gradient program was as follows: 100% B to 85% B in 30 min, 85% B to 50% B in 20 min, 50% B to 0% B in 5 min and 0% B to 100% B in 5 min. A 50 µl injection volume was used, with peaks being monitored at 280 nm and 320 nm for benzoic acid and cinnamic acid derivatives, respectively. Prior to injection, all samples were filtered through a 0.45 µm Acrodisc syringe filter from Gelman Laboratory in Michigan (Gelman Sciences, Ann Arbor, Michigan, USA). Peaks were identified based on their consistent retention times and UV spectra and then compared to the standards (0- 50 mg/L).

The pH value was determined using a digital pH meter (Hanna, HI9002; Germany). Total soluble solids (TSS) of OMWW were assessed using a digital refractometer (Atago PAL-H Master-500, Japan).

### Free phenolic content (FP)

The Folin–Ciocalteu method had been followed for FP determination^37^. Each OMWW sample (5 g) was diluted to 50 mL with distilled water. Then, 1000 µl of the obtained solution, corresponding to 100 mg of OMWW, were added to 1 mL of 10% Folin–Ciocalteu reagent. The mixture was vortexed for 2 min and the absorbance was determined after 20 min at 750 nm against the sugar analog. Gallic acid (GA) (0–200 mg/L) was used as a standard to derive the calibration curve. free phenolic content was expressed as mg of gallic acid (GA) per L of OMWW.

### Determination of total flavonoids (TF)

Total flavonoid content of OMWW was determined using the aluminum chloride colorimetric method with some modifications. 1000 µL of each OMWW sample (prepared by dissolving 5 g of OMWW in 5 mL of methanol), 300 µL of 10% aluminum chloride solution, 300 µL of 5% sodium nitrite solution and 4.3 mL of distilled water were combined together. The mixture was then incubated at room temperature for 30 min. After the incubation period, the absorbance of the resulting mixture was measured at a wavelength of 415 nm using a spectrophotometer (Shimadzu, Kyoto, Japan)^38^. A calibration curve was created using catechin (0–500 mg/L). The total flavonoid content in the OMWW was determined three times and the average result was obtained.

### Color determination of OMWW

Determination of color differences was done using a spectro-colorimeter (Tristimulus color machine Hunter Associates Laboratory, Inc., Reston, Virginia, USA) with a CIE Lab color scale (Hunter, LabScan XE, Reston, Virginia, USA), calibrated with a white standard tile of Hunter Lab color standard (LX No. 16379): *X* = 77.26, *Y* = 81.94 and *Z* = 88.14 (L* = 92.51, a* = -0.88, b* = −0.16)^39^.

### Determination of minerals and vitamins

Samples were digested in an acid solution using the Anton-Paar microwave digestion system (Multiwave PRO, Anton Paar GmbH, located in Graz, Austria). Determination of metal ions was conducted using the Agilent 5100 Synchronous Vertical Dual View (SVDV) ICP-OES, with the Agilent Vapor Generation Accessory VGA 77(Agilent Technologies, Inc., California, USA). All samples were digested to have an acceptable matrix for measuring metal ions and to provide acceptable and consistent recovery compatible with the analytical method of APHA^40^. To create an intensity calibration curve for each series of measurements, the calibration curve was composed of a blank and three or more standards obtained from Merck Company (Germany). The accuracy and precision of the metal ions measurements were approved using external reference standards from Merck, and standard reference materials and quality control samples from the National Institute of Standards and Technology (NIST, Gaithersburg, Maryland, USA) were used to confirm the instrument readings. Tocopherol (vitamin E) and vitamin A were estimated in OMWW by HPLC according to AOAC^41^ the column used was Agilent C18 (4.6 mm × 250 mm i.d.,5μm). The mobile phase was methanol: acetonitrile 65:35 and the flow rate was 1.2 mL/min. The injection volume was 20 μl for each of the sample solutions. The DAD was adjusted at 325 nm. The Fluorescence detector was adjusted at 290/330 nm (Excitation/Emission). The column temperature was maintained at 40 °C.

### Radical scavenging activity using DPPH assay

Antioxidant activity of olive mill wastewater was determined by the DPPH assay using a spectrophotometer at 517 nm. The ability of the olive mill wastewater samples to donate hydrogen atoms or electrons was measured from the bleaching of a purple-colored methanolic solution of DPPH to light yellow Goze et al. ^42^. Fifty microliters of various concentrations (200, 400, and 600 μg/mL) of olive mill wastewater in methanol, along with 4 mL of a 0.004% methanolic solution of DPPH, were added to each tube and shaken vigorously. The tubes were allowed to stand at room temperature for 30 min. Changes in absorbance of the prepared samples were measured at 517 nm. Radical scavenging activity was estimated as the inhibition percentage and calculated using the following formula:

$$\% \,{\rm{Inhibition}}=\frac{{AB}-{AA}}{{AB}}\times 100$$

Where: AB: absorption of blank sample (*t* = 0 min), AA: absorption of sample solution (*t* = 30 min).

The estimation of IC_50_ is to plot *x*-*y* and fit the data with a straight line (linear regression). IC_50_ value is then estimated using the fitted line.

### Antimicrobial activity

The antimicrobial activity of OMWW was determined using the agar well diffusion method. OMWW was tested in vitro for antibacterial activity against *Staphylococcus aureus* (ATCC:13565) and *Streptococcus mutans* (ATCC:25175) (Gram-positive bacteria), and *Escherichia coli* (ATCC:10536)*, Pseudomonas aeruginosa* (ATCC:27853), and *Klebsiella pneumoniae* (ATCC:10031) (Gram-negative bacteria) using nutrient agar medium. The antifungal activity of OMWW was tested against *Candida albicans* (ATCC:10231), *Aspergillus niger* (ATCC:22947) and *Asperagillus Ochraceous* (ATCC:22947) using Sabouraud dextrose agar medium. Ampicillin and gentamicin were used as standard drugs for Gram-positive and Gram-negative bacteria, respectively. Nystatin was used as a standard drug for fungal strains. DMSO was used as a solvent (negative) control. OMWW was tested at a concentration of 15 mg/mL against both bacterial and fungal strains.

Sterilized media were poured into sterilized Petri dishes (20–25 mL per dish) and allowed to solidify at room temperature. To prepare the microbial suspension, sterilized saline was used to achieve a McFarland 0.5 standard solution (1.5 × 10^^5^ CFU/mL). The turbidity of the suspension was adjusted to OD = 0.13 using a spectrophotometer at 625 nm. Within 15 min of adjusting the turbidity, a sterile cotton swab was dipped into the suspension and spread onto the dried agar surface. The dish was then covered and allowed to dry for 15 min. Wells of 6 mm diameter were created in the solidified agar using a sterile borer. Each well was filled with 100 µL of the tested compound solution using a micropipette. The plates were incubated at 37 °C for 24 h in the case of antibacterial activity^43^. This experiment was carried out in triplicate, and zones of inhibition were measured in mm.

### Preparation of enriched vegetable oils (corn and soybean oils) with dried OMWW

Two different types of oil were enriched with dried OMWW: The OMWW had been dried by adding water and glycerol (1:1), then the oil was added up to a total volume of 1000 mL. The bottle was shaken well and the samples were stored in the freezer for further analysis.

### Determination of oils oxidative stability (corn and soybean oils) was done by Rancimat

According to Oleynikov^44^. This test was used to determine the antioxidant activity of OMWW added at different levels of (200, 400 and 600 mg/L) to corn oil and soybean oil. These were compared to the effect of BHT at (200 mg/L) and pure corn oil and soybean oil (served as a control). The induction times of all the treated samples as well as the controls were calculated and recorded. The antioxidant activity (AA) and increasing index % were calculated from the measured induction times, as follows:

$$\mathrm{Antioxidant\; activity}\,(\mathrm{AA})=\frac{\mathrm{Ind}.\mathrm{time\; of\; oil\; with\; antioxidant}}{\mathrm{Ind}.\mathrm{time\; of\; control}}$$

$${\rm{Increasing\; Index}} \% =\frac{{IP\; with\; antioxidant}-{IP\; of\; the\; control}}{{IP\; of\; the\; control}}\times 100$$

Where IP: induction period

### Preparation of French fries

Deep frying was performed according to ref. ^32^, with slight modifications as follows: both oils enriched with and without dried OMWW (the frying pan was filled with 1000 mL + 10 mL) were used for the process of frying potatoes (200 + 0.5 g) over two successive frying sessions (for 10 min each time) without replenishment, under domestic frying conditions (oil temperature reached 180 °C). French fries made from potatoes that were cut into 1/2-inch pieces and weighed approximately 200 + 0.5 g, were placed into the frying oil. Each batch of potatoes was deep-fried for a duration of 10 min without the need for additional oil. The cooking oil was allowed to cool for approximately 30 min before each new frying operation. Excess oil on the French fries was allowed to drain for 5 min on a cross-linked steel wire mesh and then the food was cooled on a kitchen paper sheet.

### Preparation of mayonnaise

The recipe followed for mayonnaise preparation was done according to ref. ^45^. The primary composition of mayonnaise includes 70-80% fat, a pH range of 3.2-4.2, a water activity of 0.95, and acetic acid content ranging from 0.8% to 6.0%. Control sample recipe of the present study contained the following ingredients in percentage (w/w): oil 70%, fresh egg yolk 22.17%, vinegar 0.63%, sugar 0.63%, salt 1.26%, mustard powder 0.31%, white pepper 0.31% and lemon juice 2.20%. The enriched mayonnaise samples were prepared by adding OMWW (1 and 3%), another sample was prepared by adding TBHQ (200 mg/L for comparison). The prepared, samples were kept into glass jars with screw caps and then were labeled, covered and stored at room temperature (25 ± 1°C) for 5 weeks.

### Organoleptic evaluation of French fries and mayonnaise

Sensory evaluation was carried out by 10 trained panelists (six males and four females, aged 30–55 years) from the staff members in food science lab of Nutrition and Food Science Department, Faculty of Home Economics, Helwan University, Cairo, Egypt. The research ethics have been followed**:** investigators of the present study took all necessary precautions to minimize risks to participants, especially regarding allergies or food intolerances. They informed of their right to withdraw from the study at any time without penalty and maintaining the confidentiality of all participants’ data. A 5-point score test was used according to Stone et al. ^46^ for these characteristics (appearance, odor, taste, color, tenderness and general acceptability for French fries) and (odor, taste, flavor, color, degree of cohesion, degree of smoothness and general acceptability for mayonnaise).

### Determination of free phenolic content of French fries and mayonnaise

Free phenols content were determined in French fries and mayonnaise using the Folin–Ciocalteu reagent according to ref. ^47^. The test is based on all phenolic compounds contained in the samples being oxidized by the Folin–Ciocalteu reagent. Free phenolic content was expressed as mg of gallic acid (GA) per 100 g of sample.

### Thiobarbituric acid value (TBA) of French fries and mayonnaise

The method was carried out according to ref. ^48^, 1g of sample was dissolved in 3.5 mL of cyclohexane and 4.5 mL of 7.5% trichloroacetic acid (TCA)/0.34% TBA was subsequently added. The resulting mixture was shaken for 5 min. After centrifugation for 15 min at 2870 g, the TCA-TBA phase was removed and heated in a water bath at 100 °C for 10 min; absorbance measured at 532 nm. A calibration curve was constructed using serial dilutions (0.808–12.92 nmol/mL) of 1,1,3,3-tetramethoxypropane in water, which hydrolyzes to produce malondialdehyde (MDA). The TBARS content, expressed as MDA, was determined from the standard curve. Product quality was also evaluated weekly during a storage period of 2 weeks at room temperature 28 °C.

### Microbiological examination of mayonnaise

All samples were analyzed for aerobic plate count and yeast and mold; all analysis was performed by using the standard procedures outlined in ISIRI^49^.

### Color determination of mayonnaise

Color differences were measured for supplemented and unsupplemented sample by using a spectro-colorimeter (Tristimulus color machine, Hunter Associates Laboratory, Inc., Reston, Virginia, USA.) with CIE lab color scale (hunter, lab scan XE, Reston Virginia, USA.) calibrated with a white standard tie of Hunter Lab color standard (LX No. 16379): *X* = 77.26, *Y* = 81.94 and *Z* = 88.14 (L* = 92.51, a* = −0.88, b* = −0.16) as the method of ref. ^39^ as follows: Subcript (“0” hue) Indicates color of control, Hue angle (tan^−1^ *b*/*a*) and saturation index [$\sqrt{{a}^{2}+{b}^{2}}$] were also calculated.

### Proximal composition and nutritive value determination of mayonnaise

The prepared mayonnaise samples were subjected to chemical analysis to determination their proximal composition: Ash, total protein, total fat, total carbohydrates (by difference) according to AOAC^41^.

### Determination of minerals and vitamins

The prepared mayonnaise samples which achieved better characteristics were subjected to determination for vitamin E by HPLC (Agilent Technologies Inc., California, USA). Also, products achieved better characteristics were subjected to minerals analysis for calcium (Ca) and potassium (K) according to AOAC^41^.

### Statistical analysis

Statistical analyses were performed in triplicate, except for the sensory evaluation results, which was conducted with ten replicates, using SPSS V.15.0 for Windows (IBM, USA). One-way ANOVA was carried out at (*p* < 0.05).

## Supplementary information

Supplementary Information

## Data Availability

The data supporting this study's findings are available from the corresponding author (RJ) upon reasonable request.
