# Supplementary material for: Improving stability of frying oils and food quality with addition of dried olive mill wastewater
Source: NPJ Sci Food. 2025 May 16;9:75. doi: 10.1038/s41538-025-00430-x (PMC12084610; doi:10.1038/s41538-025-00430-x)
Supplement: Supplementary file 1 — Supplementary Information [file 41538_2025_430_MOESM1_ESM.pdf]

Supplementary information

Supplementary data include: stages for extracted oil and by-products of olive fruit, polyphenolic composition of olive mill waste water dried by using HPLC, HPLC chromatograph of tocopherol vitamin (E) and A vitamin in OMWW, and photograph image of mayonnaise prepared supplemented with different percentages of dried OMWW.

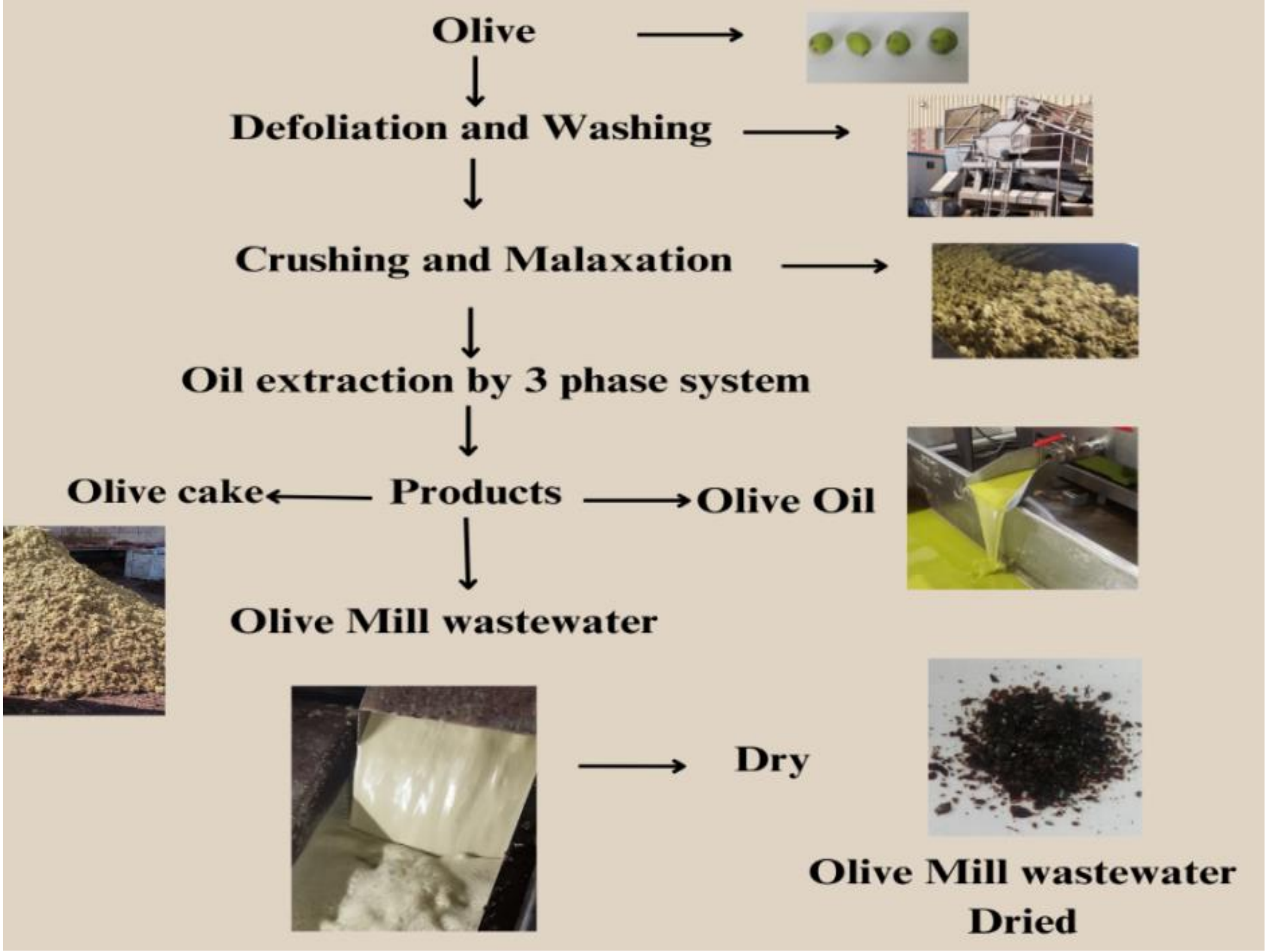

Supplementary Fig. 1 Stages for extracted oil and by-products of olive fruit.

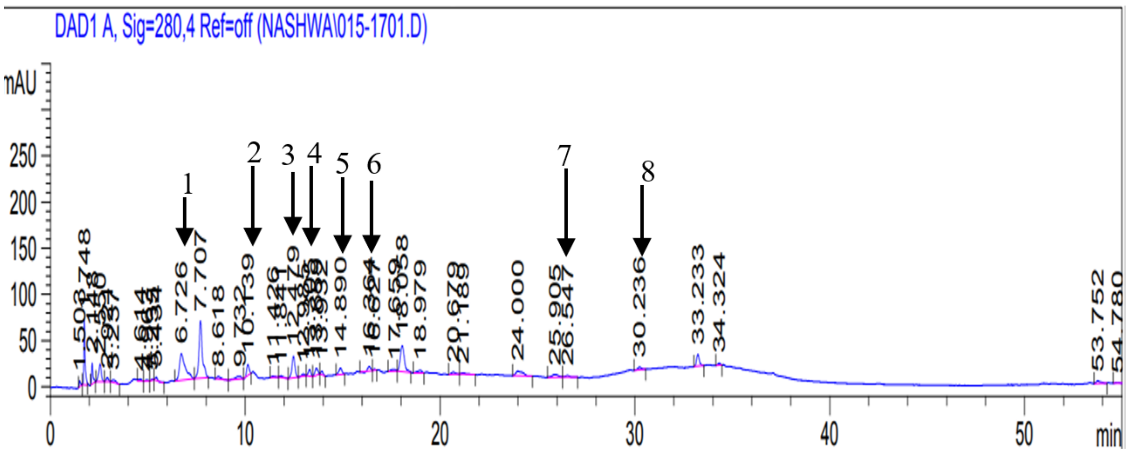

Supplementary Fig. 2 Polyphenolic composition of olive mill waste water dried by using HPLC. Protocatechuic acid (1), *p*-hydroxybenzoic acid (2), Chlorogenic acid (3), Caffeic acid (4), Syringic acid (5), Vanillic acid (6), p-coumaric acid (7), Oleuropein (8).

Supplementary Table 1 Polyphenolic composition of olive mill waste water dried by using HPLC

Compound	Retention time (min)	Concentration (mg/g)
Oleuropein	33.233	2.351704
Protocatechuic acid	7.707	0.65947
p-hydroxybenzoic acid	10.13	0.102071
Chlorogenic acid	12.47	0.077954
Syringic acid	14.89	0.051573
Caffeic acid	13.65	0.037550
p-coumaric acid	26.5	0.018707
Vanillic acid	16.36	0.014242

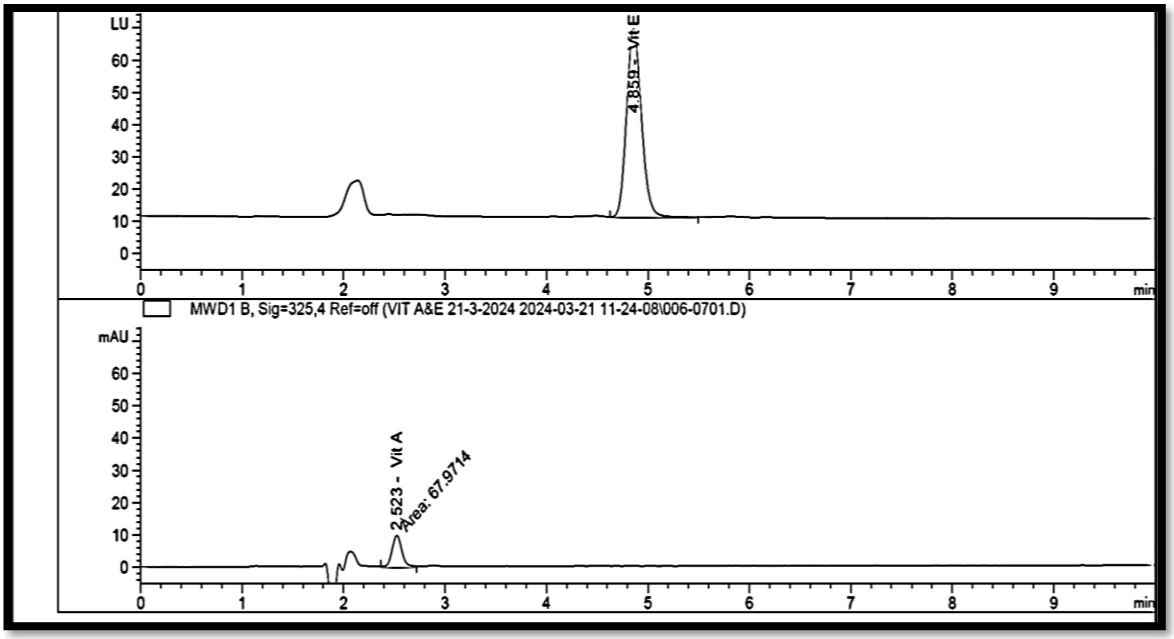

Supplementary Fig. 3 HPLC chromatograph of tocopherol vitamin (E) and A vitamin.

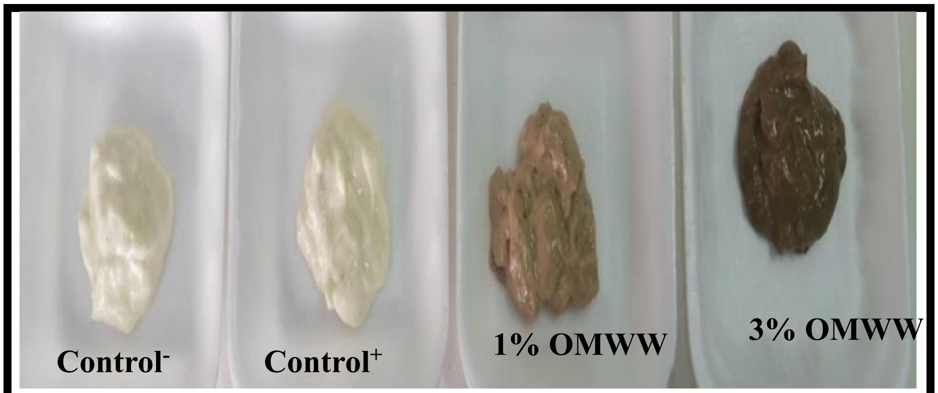

Supplementary Fig. 4 photograph image of mayonnaise prepared supplemented with different percentages of dried OMWW.
